# The clinical potential of blood-proteomics in multiple sclerosis

**DOI:** 10.1186/1471-2377-13-45

**Published:** 2013-05-21

**Authors:** Roberto De Masi, Sergio Pasca, Rocco Scarpello, Adele Idolo, Antonella De Donno

**Affiliations:** 1 Laboratory of Neuroproteomics, Multiple Sclerosis Centre, Complex Operative Unit of Neurology-Stroke Unit, “F. Ferrari” Hospital via circonvallazione, 73042, Casarano, Lecce, Italy; 2 Complex Operative Unit of Neurology-Stroke Unit, “F. Ferrari” Hospital, Casarano, Lecce, Italy; 3 Multiple Sclerosis Center, “F. Ferrari” Hospital, Casarano, Lecce, Italy; 4 Department of Biological and Environmental Sciences and Technologies, Lab of Hygiene, University of Salento, Lecce, Italy

**Keywords:** Multiple sclerosis, Blood-proteomics, Interferon therapy

## Abstract

**Background:**

The aetiology of multiple sclerosis (MS) remains unknown. This hampers molecular diagnosis and the discovery of bio-molecular markers. Consequently, MS diagnostic procedures are complex and criteria for assessing therapeutic efficacy are controversial, suggesting that a pathophysiological rather than an aetiological approach to the disease would be more appropriate. In this regard, blood-proteomics represents a still-unexplored tool. We investigated the potential of proteomics as applied to peripheral blood mononuclear cells (PBMCs) for differentiating treatment-naive RR-MS patients from healthy controls and from IFN-treated RR-MS patients.

**Methods:**

A comparative analysis of PBMC proteins isolated from 13 unselected IFN-treated RR-MS patients, 6 IFN-untreated RR-MS patients and 14 matched healthy controls was performed using two-dimensional gel electrophoresis and MALDI-TOF mass spectrometry. We considered the volume of each spot, expressed as a percentage of the total volume of all spots in the gel. Heuristic clustering was applied to a composite population made up of a random sequence of gels from the different groups in comparison. For the differentially expressed proteins, we applied the Student's *t*-test to identify only those down- or up-regulated at least 2.5-fold [Ratio(R) ≥ 2.5] with respect to the homologous spots of the compared groups.

**Results:**

Rho-GDI2, Rab2 and Cofilin1 were found to be associated with down-regulated and naïve group phenotypes; Cortactin and Fibrinogen beta-Chain Precursor were found to be associated with down-regulated and group-related IFN-treated RR-MS phenotypes. Thus, by means of similarity analysis, the proteomes were homogeneously segregated into three distinct groups corresponding to naive, IFN-treated and healthy control subjects. Interestingly, no separation was found between IFN-treated and healthy controls. Moreover, the molecular phenotypes were consistent with disease pathogenesis.

**Conclusions:**

We demonstrated for the first time, albeit only with preliminary data, the aprioristic possibility of distinguishing naive and IFN-treated MS groups from controls, and naive from IFN-treated MS patients using a blood sample-based methodology (i.e. proteomics) alone. The functional profile of the identified molecules provides new pathophysiological insight into MS. Future development of these techniques could open up novel applications in terms of molecular diagnosis and therapy monitoring in MS patients.

## Background

Despite the extensive literature in the aetiological field, infectious and genetic theories have failed to identify the cause of the disease, and so multiple sclerosis (MS) is an autoimmune pathology whose aetiology is still unknown [1,2]. This is responsible for the lack of molecular diagnoses and therapy monitoring. On the other hand, there is increasingly consistent pathogenetical evidence that peripheral auto-reactive T-cells play a central role in provoking inflammatory demyelination and axonal loss in the brain parenchyma [3]. In addition, by acting on peripheral T-cells, interferon-beta (IFN-β) is thought to reduce disease activity, with an MRI-detectable effect in relapsing–remitting multiple sclerosis (RR-MS) patients [4-6], confirming the critical role of peripheral blood mononuclear cells (PBMCs) in CNS damage [7].

Molecular mimicry and epitope spreading further complicate aetiological investigation, suggesting that a pathophysiological rather than an aetiological approach to MS diagnosis may be more appropriate. Consequently, the MS-patient is diagnosed by pathophysiological evaluation of clinical or paraclinical dissemination in time and space [8-10], and the only biological tests currently considered to be of diagnostic relevance are oligoclonal band assessment and the exclusion of MS-mimicking conditions. Recently, proteomics has been successfully used to study autoimmune diseases by contextualizing the pathophysiological status of target cells (or tissues) with reference to their protein expression profile, the so-called molecular phenotype. This pathophysiology-based point of view may allow MS to be studied regardless of its aetiology. Dotzlaw H. and Schulz M. applied this technique to the PBMCs of rheumatoid arthritis patients and identified a differential fingerprint that separated diseased from healthy control subjects [11,12]. In addition, the resulting differentially expressed proteins helped to elucidate the molecular mechanisms of the disease. Blood-proteomics is also able to differentiate patients with Alzheimer’s disease (AD) from healthy controls by providing a panel of plasma-proteins that also predicts progression to AD in preclinical patients affected by mild cognitive decline [13].

More recently, we found a functional correlation in MS patients between brain atrophy and the protein expression profile of PBMCs, confirming their pathophysiological involvement in disease evolution [14]. Despite the encouraging findings, to our knowledge, there has been no attempt to electively elucidate the diagnostic potential of PBMC-based proteomics in MS.

We assessed the potential of PBMC-based proteomic analysis applied to a random, blind population made up of IFN-untreated MS patients and healthy controls, differentiating and separating them into homogeneous groups. Secondarily we investigated the ability of the same methodology to blindly differentiate IFN-treated RR-MS patients from IFN-untreated (naïve) patients.

## Methods

### Study population

Three different classes of study population subjects were enrolled in a case-controlled cross-sectional proteomic study at the MS unit of “F. Ferrari” Hospital, Neurology Division, Casarano (Le), Italy:

1) Thirteen (seven male, six female) consecutive unselected IFN-treated RR-MS (RRt) patients taking 30 μg of interferon β-1a I.M. a week in accordance with the international guidelines of Jacob et al. [15].

2) Six (three male, three female) consecutive sex/age matched IFN-untreated RR-MS (RRu, naïve) subjects.

All MS patients were previously diagnosed in accordance with standard criteria [10] and gave blood samples following a steroid- and exacerbation-free period of at least three months. Patients were selected for the study on the basis of the following criteria: (i) they were in the RR phase of the disease, classified in accordance with standard disease course criteria [16]; (ii) they were aged between 23 and 65; (iii) they had not undergone any immune-modulation therapy (naïve patients); (iv) they were prepared to express written informed consent. Patients who had been treated with immunosuppressive drugs or were affected by other comorbidity conditions were excluded from this study. Only non-interfering symptomatic therapy was tolerated.

3) Fourteen (seven male, seven female) age/sex matched healthy controls (HC).

All subjects were enrolled and underwent blood sampling after expressing informed written consent. Approval of this study was provided by the local hospital and by the ethics committee of the Local Health Authority of Lecce (ASL/LE).

### PBMC isolation and lysis

Heparinized blood (15 ml) was collected by venipuncture and immediately processed. First, blood was centrifuged at 150 × g for 5 min and the platelet-enriched supernatant was removed. The pellet was then diluted with PBS (Phosphate Buffered Saline) solution and layered on a Ficoll density gradient (GE Healthcare). Subsequently, PBMCs were isolated by centrifugation (400 × g for 40 min) and processed within 1h. After isolation, the lympho-monocytes were washed twice in PBS and lysed in 0.5 ml of sample buffer (CHAPS) and protease inhibitor cocktail. To minimize nucleic acid interference with protein migration, the sample was sonicated 3 times x 5 sec on ice (low amplitude). Finally, protein concentration was determined by Bradford’s method (Bio-Rad Protein assay).

### Two-dimensional gel electrophoresis

#### First dimension run

##### Sample loading

80 μg of proteins were mixed with rehydration solution (7 M Urea, 2 M thiourea, 4% CHAPS, 50 mM DTT and 0.8% IPG Buffer) to obtain a final volume of 250 μl. The samples were incubated for 30 min at 4°C and applied to 13 cm IPG pH 4–7 gel strips (Amersham Biosciences).

##### Isoelectric focusing (IEF)

IEF was performed with an IPGphor system (GE Healthcare) in 7 steps by gradually increasing the voltage after rehydration for 4 h: 30 V for 12 h, from 30 V to 500 V in 30 min, 500 V for 1 h, from 500 V to 1000 V in 30 min, 1000 V for 1 h, gradient from 1000 V to 8000 V in 30 min, 8000 V for 2 h, making a total of 20,617 Vh.

##### Equilibration

after IEF, the strips were equilibrated in 6 M Urea, 50 Mm Tris–HCl (pH 8.8), 30% glycerol, 2% SDS and 10 mg/ml DTT for 18 min, followed by 25 mg/ml iodoacetamide for 10 min.

### Second dimension run

The second dimension was performed on a 9-16% gradient SDS-polyacrylamide gel (16 × 18 cm, 1.5 mm thick) and run at a constant current of 28 mA/gel at 8°C until the BPB reached the anodic end of the gel. Wide range protein standard (Sigma-Aldrich) was loaded together with the sample.

#### Development and staining

proteins were visualized by ammoniacal silver staining, adding thiosulfate after the gel polymerization step [17]. Each sample was run in duplicate, making a total of sixty-six gels subjected to 2-D electrophoresis.

### Image and statistical analysis

Gel images were acquired using an Image Scanner (GE Healthcare), with a scanning resolution of 300 dpi. Spot detection, matching and data analysis were performed with Image MasterTM 2D Platinum software, version 5.0 (GE Healthcare). The gels were divided into three classes (healthy controls, IFN-untreated RR-MS patients and IFN-treated RR-MS patients) and a reference gel with the highest number of spots was created for each class. The software was then used to detect and match spots between gels by the matching method included in the software package. Spot detection and spot matches were corrected manually where necessary. The analysis was carried out by comparing the volume of each spot expressed as a percentage of the total volume of all spots in the gel.

Heuristic clustering was used on a random population composed of a blind sequence of gels from the classes in comparison. This analysis assigned each gel to a homogenous subclass depending on the similarity between each spot and its homologue in all the other gels in comparison. The Student’s *t*-test was applied to the differentially expressed proteins in order to identify only those down- or up-regulated at least 2.5-fold [Ratio(R) ≥ 2.5] with respect to homologous spots of the compared groups. In addition, Leven’s test was applied to every differentially expressed spot to assess the similarity of variance between the compared groups.

Cluster analysis was applied first to a composite population made up of naïve patients and healthy controls, and then to a composite population made up of IFN-treated and untreated RR-MS patients. In both cases we used a random blind sequence of gels.

### Protein identification

For preparative mass spectrometry analysis, gels were stained with MS-compatible silver staining or with Blue Silver colloidal Coomassie [18,19].

Silver stained spots were excised from preparative gels and destained with hydrogen peroxide and ammonium bicarbonate as reported by Sumner et al. [20]. Coomassie stained spots were processed by incubating with 50 μl of 50 mM NH_4_HCO_3_ in 40% ethanol until full decolouration. The spots were then incubated with 50 μl of acetonitrile for 10 min. The dried gel pieces were rehydrated in 16 μl (20 μg/ml) of trypsin solution (Sigma) and digested overnight at 37°C.

The peptide extract was desalted and concentrated by reverse phase extraction using ZipTip C18 microcolumn (Millipore, CA, USA). Peptides were directly eluted with 2 μl of α-cyano-4-hydroxycinnamic acid in 33% acetonitrile, TFA 0.1%, on a ground steel MTP 384 target (Bruker Daltonics).

MALDI-TOF analyses were performed on a Reflex IV mass spectrometer (Bruker Daltonics) in reflectron positive ion mode after internal and external calibration of the spectrum. The MALDI mass spectrum was externally calibrated with the peptide standard calibration II mixture (Bruker Daltonics) and then internally calibrated with trypsin autodigestion products (842.51 m/z, 1045.56 m/z and 2239.14 m/z). Analyses were carried out in the mass range from 700 to 4000 m/z and about 300 laser shots were taken for each spectrum.

Database searches were conducted with the MS-Fit or Mascot programs against Swiss-Prot and NCBI databases with the following parameters: human species, mass tolerance of 50 ppm, one missed cleavage site for tryptic peptides, allowing the modification of carbamidomethylation (C) and variable oxidation (M).

## Results

**Figure 1:**
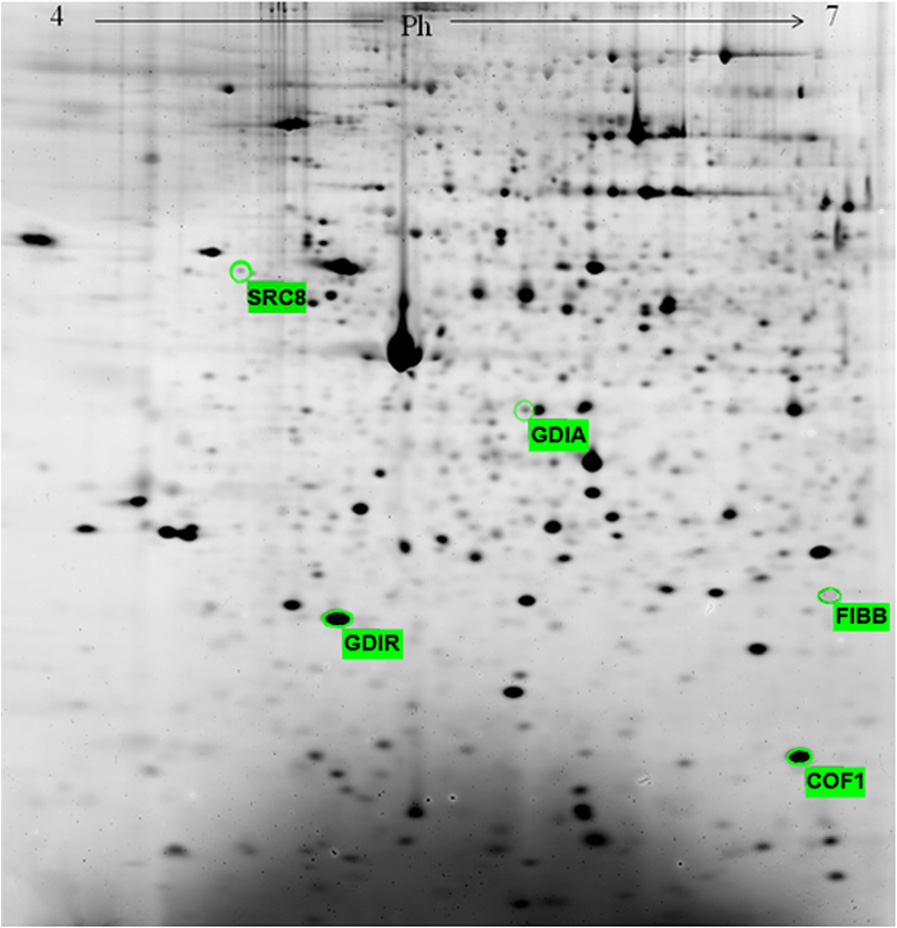
**Reference map of PBMCs.** Spots showing differential expression are indicated.

Demographic characteristics were comparable in the diseased and control groups (Table 1). The mean duration of IFN-based therapy at the start of the study was 7 years. Figure 1 shows a reference map of PBMCs where all the spots showing differential expression are indicated. A list of them with their Mascot identification data is shown in Table 2.

**Table 1:** **Clinical and demographic features of treated MS** (**RRt**), **untreated MS** (**RRu**) **and healthy control** (**HC**) **subjects**

	**RRt (n = 16)**			**HC (n = 12)**			**RRu (n = 6)**			***p value***
	**range**	**mean**	**SD**	**range**	**mean**	**SD**	**range**	**mean**	**SD**	
DISEASE DURATION	2.0-14.0	7.43	4.5				2.9-13.9	7.83	3.9	*p* = *0*.*15*
AGE AT STUDY	23.0-65.0	42.6	11.1	22.2-64.3	41.8	10.6	20.9-66.0	42.9	12.2	*p*^*1*,*2*,*3*^ ≥ *0*.*22*
EDSS	0.0-5.0	2.0	1.7							
SEX RATIO (♀/♂)	56%/44%			58%/42%			57%/43			

*p*^*1*,*2*,*3*^: *p* value of comparison between HC/RRt, RRu/RRt, RRu/HC groups respectively.

**Table 2:** Identified proteins and corresponding Mascot score

**Entry name**	**Peptide matches**	**Swiss**-**Prot AC number**	**Description**	**pI**	**Mw**	**pI**	**Mw**	**Coverage**	**Score Mascot**
COF1	8	P23528	Cofilin 1 (non-muscle isoform)	8.3	18371	5.91	19	57%	107
SRC8	22	Q14247	Src substrate cortactin (Amplaxin)	5.2	61637	5.08	85	35%	207
FIBB	31	P02675	Fibrinogen beta chain precursor	8.5	55929	6.03	42	54%	227
GDIR	10	P52565	Rho GDP-dissociation inhibitor 1	5.0	23076	4.96	26	39%	88
GDIA	25	P31150	Rab GDP dissociation inhibitor alpha	5.0	50583	4.99	59	52%	304

**Figure 2:**
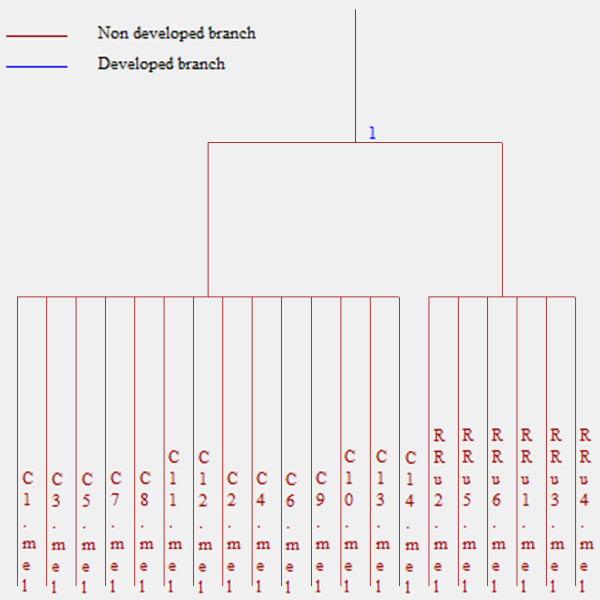
**Dendrogram indicating complete separation between gels from healthy controls and IFN**-**untreated patients.**

**Figure 3:**
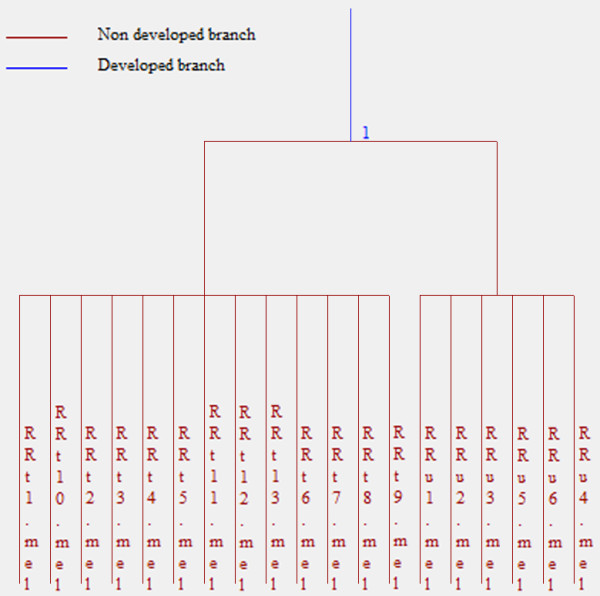
**Dendrogram indicating complete separation between gels from IFN**-**untreated and IFN**-**treated patients.**

The heuristic clustering dendrogram indicated complete separation between the gels of healthy controls and those of diseased IFN-untreated patients (Figure 2); complete separation was also obtained between the gels of IFN-untreated and IFN-treated patients (Figure 3). These results were obtained by applying cluster analysis to one population composed of a random blind sequence of all the gels of HC and RRu subjects and another composed of all the gels from the RRu and RRt patients.

**Figure 4:**
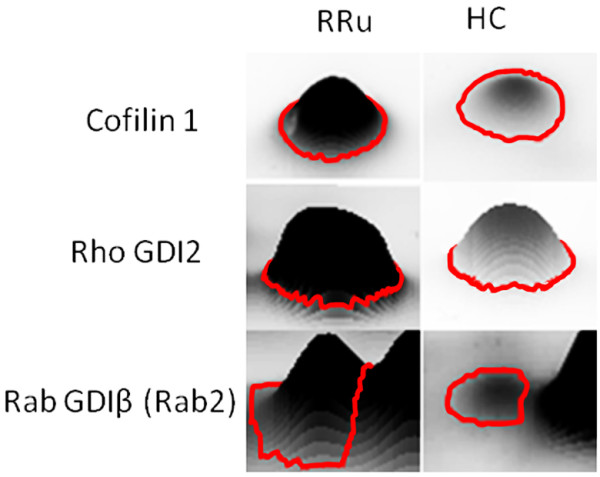
Shape of most representative spots.

In the first case, the differentially expressed proteins were: Rho-GDI2, Rab GDIβ (Rab2) and Cofilin 1. In the second case, the differentially expressed proteins were: Cortactin and the Fibrinogen β Chain Precursor. Specifically, Rho GDI2, Rab2 and Cofilin were found to be down-expressed in IFN-untreated patients in comparison to healthy controls, while Cortactin and Fibrinogen β chain precursor were found to be up-regulated in the untreated group compared to the treated group. Moreover, equality of variance was found in all the compared groups, except for Cofilin between the IFN-treated and untreated groups (R and Leven’s tests in Table 3). Figure 4 shows the shape of the most representative spots.

**Table 3:** **Differentially expressed proteins with** % **volume and related statistical parameters of treated MS** (**RRt**), **untreated MS** (**RRu**) **and healthy control** (**HC**) **subjects**

**Entry name**	**Average Vol% RRu (SD; range)**	**Average Vol% HC (SD; range)**	**Average Vol% RRt (SD; range)**	**RRu change with respect to HC (Vol% ratio; *p *)**	**RRu change with respect to RRt (Vol% ratio; *p *)**	**Levene’s test for equality of variance (RRu/HC)**	**Levene’s test for equality of variance (RRt/RRu)**
**Rho GDI2**	0.7340 (0.26; 0.60)	1.9738 (0.28; 0.95)	//	Down 2.8; (*p* = *0*.*000*)	//	n.s.	//
**Rab GDIβ** (**Rab2**)	0.2883 (0.04; 0.12)	0.0976 (0.05; 0.19)	//	Down 2.9; (*p* = *0*.*000*)	//	n.s.	//
**Cofilin 1**	0.1946 (0.10; 0.24)	0.9651 (0.48; 1.83)	//	Down 4.9; (*p* = *0*.*000*)	//	*p* = *0*.*043*	//
**Cortactin**	0.3912 (0.04; 0.11)	//	0.0546 (0.04; 0.13)	//	Up 7.1; (*p* = *0*.*000*)	//	n.s.
**Fibrinogen β Chain Precursor**	0.1372 (0.02; 0.08)	//	0.0039 (0.01; 0.03)	//	Up 35.1; (*p* = *0*.*000*)	//	n.s.

n.s.: not significant.

Lastly, clustering analysis applied to a population made up of a random blind sequence of all the gels from the RRt patients and HC subjects showed no homogeneous class separation.

All the differentially expressed spots sequenced by MALDI-TOF analysis exhibited R ≥ 2.5 (*p* ≤ *0*.*001*).

## Discussion

This study assessed the aprioristic potential of proteomics for discriminating between RR-MS subjects and healthy controls and between IFN-treated RR-MS patients and untreated patients. Blind heuristic clustering applied to a random population of PBMC gels from IFN-treated and untreated MS patients was able to separate treated from naïve subjects and the latter from healthy controls. Specifically, Rho-GDI2, Rab GDIβ (Rab2) and Cofilin 1 were found to be down-regulated in naïve patients, and Cortactin and Fibrinogen β Chain Precursor were found to be down-regulated in the IFN-treated group. Each group of gels belonging to a dendrogram branch was separated from the other branches by the presence of differentially expressed spots exhibiting two key features: the non-overlapping mean % volume values and the large difference between the mean values (at least two and a half times the mean absolute value) of the % volume of each sub-group.

Although these findings represent preliminary data, they demonstrate for the first time the concrete role of proteomics in the molecular diagnosis of MS. Moreover, proteomics is able to distinguish the PBMC profile of an IFN-treated patient from that of a naïve one. This latter finding may seem to be of low importance, but it could contribute to the molecular characterization of patients who do not respond to interferon therapy.

In addition, our data confirmed, at a molecular level, the disregulation and consequent diversion of the immune system to the “self” in MS patients. We also found quantitative rather than qualitative differences between the PBMC profile of naïve patients and that of healthy controls. This is in line with our previous work, which did not find a differential pattern in MS. The application of cluster analysis in the present study is seen to have a diagnostic value based on a pathophysiological methodology, and the differentially expressed proteins identified here exhibit a coherent functional profile in the pathophysiological context of MS.

Rho-GDI2 and Rab GDIβ (Rab2) are key GTPase inhibitors: they regulate a group of high-level proteins in cell biology and are involved in various fundamental cellular events including secretion, proliferation, intracellular signalling, cytokinesis, vesicular trafficking and lymphocyte extravasation through blood-CNS barriers.

Three general classes of GTPase signalling regulators have been identified: guanine nucleotide exchange factors (GEFs), GTPase-activating proteins (GAPs) and guanine nucleotide dissociation inhibitors (GDIs).

Rho-GDI proteins form a large complex, with the Rho protein helping to prevent diffusion within the membrane and the cytosol [21]. Rho GTPases at the plasma membrane are in turn known to be associated with TCR cascades following antigen recognition in the presence of MHC molecules. This association is developed by the ZAP-70 protein. A substrate for its activity is the adapter molecule LAT (linker for activation of T cells), linked to the Rho/Rac domain in a multi-protein signalling complex that includes PLCγ, Grb2 and Ras. They are the main effectors for intracellular actin dynamics and gene expression. Interestingly, there is much evidence to indicate that Rho GTPase is a critical signalling intermediate in vascular inflammation and inflammatory injury.

Rab2 proteins are monomeric GTPases specializing in vesicular trafficking, as their sub-cellular localization in the cis-Golgi lattice suggests. Moreover, they can interact with the SNARE system, thereby coupling membrane cohesion to their fusion.

It thus seems clear that GDI represents a pivotal regulator of the major cellular functions that support the same abnormal mechanisms postulated in MS pathogenesis. The GDI down-expression in the PBMCs of naïve MS patients is evidence for the basal inflammatory activity of leukocytes, as expected in a chronic inflammatory disease. Consistently, GDI down-expression was not seen in the IFN-treated MS group. Indeed, the anti-inflammatory effects of β-interferon, with notable reduction in cell trafficking, secretion, proliferation and leukocyte extravasation, are well known. Likewise, Cofilin, a ubiquitous eukaryotic actin-binding protein with actin-severing activity, was seen to be down-expressed in the IFN-treated group.

The down-expression of Cortactin and the Fibrinogen β Chain Precursor in the treated MS-group again suggests a pharmacological effect. Indeed, the former, which is over-expressed in human carcinoma cells, is implicated in the modulation of cellular adhesion; the latter is an extracellular protein that stimulates leukocyte functional activity and adhesion during inflammation [22-24].

Thus we argue that naïve RR-MS is characterized by chronic, basal activation of the fundamental pro-inflammatory mechanisms correlated with Rho and Rab2 GDI de-inhibition. This condition does not seem to characterise IFN-treated patients, as also suggested by the decrease in Cortactin and Fibrinogen beta chain precursor in the RRt group.

In the literature, the main proteomic results have been obtained in neoplasmic, genetic and infectious diseases that are characterized by aberrant molecular phenotypes such as disease-specific differentially expressed proteins. In our study, rather than aberrant phenotypes we found aberrant expression of normal proteins. Our differentially expressed proteins thus do not appear to indicate pathological specificity, but reflect the functional condition of PBMCs.

One weakness of our study is the limited population, but two other elements need to be considered: with the sole exception of Cofilin, the low and homogenous magnitude of inter-group and intra-group data dispersion confirms the accuracy of the method; the difference in protein expression over the third standard deviation makes the study fairly robust.

## Conclusions

Although our statements refer to a population already screened for other MS-like conditions, we can conclude that PBMC proteomics coupled with heuristic clustering is able to distinguish treatment-naïve MS patients from healthy control subjects and IFN-treated patients from untreated patients.

Lastly, Rho- and Rab2-GDI inhibitors, Cofilin, Cortactin and the Fibrinogen beta chain precursor can improve our knowledge of the pharmacodynamics of β-interferon and the pathophysiology of MS. Although further confirmation by means of routine-based techniques is warranted, they should be considered as potentially clinically relevant signalling factors in the laboratory criteria of IFN-responding RR-MS patients and in the early diagnosis of the disease.

## Abbreviations

MS: Multiple sclerosis; PBMCs: Peripheral blood mononuclear cells; IFN-β: Interferon-beta; RR-MS: Relapsing–remitting multiple sclerosis; AD: Alzheimer’s disease; RRt: RR IFN-treated; RRu: RR IFN-untreated; HC: Healthy controls; PBS: Phosphate Buffered Saline; IEF: Isoelectric focusing; GEFs: Guanine nucleotide exchange factors; GAPs: GTPase-activating proteins; GDIs: Guanine nucleotide dissociation inhibitors.

## Competing interests

The Authors declare that they have no competing interests.

## Authors’ contributions

RDM was involved in study design, data acquisition, data analysis, manuscript drafting, literature research, statistical analysis and clinical studies. SP was involved in study design, data acquisition, data analysis, manuscript drafting, literature research and clinical studies. RS was involved in study design, data acquisition, data analysis, manuscript drafting, literature research and clinical studies. AI performed data acquisition and literature research. ADD drafted the manuscript and performed literature research. All authors read and approved the final manuscript.

## Pre-publication history

The pre-publication history for this paper can be accessed here:

http://www.biomedcentral.com/1471-2377/13/45/prepub
